# Helminth infections in light of an ongoing intervention in endemic areas of Guragae zone, Southern Ethiopia: an implication for neglected tropical diseases elimination in Ethiopia by 2020

**DOI:** 10.1186/s40794-019-0083-y

**Published:** 2019-05-03

**Authors:** Teha Shumbej, Tadele Girum

**Affiliations:** 1 Department of Medical Laboratory Sciences, College of Medicine and Health Sciences, Wolkite University, Wolkite, Ethiopia; 2 Department of Public Health, College of Medicine and Health Sciences, Wolkite University, Wolkite, Ethiopia

**Keywords:** Helminths, School-aged children, Guragea zone, Ethiopia

## Abstract

**Introduction:**

Helminth infections are among the major public health problems in developing countries. Considerable efforts have been made towards the control of morbidity caused by infection with helminths in Ethiopia. The national control program is designed to achieve the elimination of helminth infections as a major public health problem by 2020.

**Objective:**

The objective of this study was to determine the current status and infection intensity of helminths in the endemic area of Guragae zone, Southern Ethiopia.

**Methods:**

An institutional based cross-sectional study was carried out between April and June 2017 in Gurage zone. School-aged children (SAC) were selected using a multistage sampling method and invited to participate in the study. Parasitological test examination was done using the Kato-Katz technique in Wolkite University parasitology laboratory. SPSS version 21 was used for data management and analysis.

**Results:**

A total of 597 (98% compliance rate) participants were able to provide complete data. The study revealed that 21.6% (129/597) SAC were infected with one or more species of helminth. *S. mansoni* was the most prevalent helminth (12.9%) followed by hookworms (4.3%). The overall infection intensity expressed as geometric mean for *A. lumbricoides*, *T. trichiura*, hookworms, and *S. mansoni* were 301, 31,103, and 158 eggs per gram of stool, respectively. The multivariable logistic regression model estimated that being in the age group of 5–9 years (AOR = 1.43, 95% CI 0.4–0.9), washing raw food and vegetables using river water (AOR = 2.4, 95% CI 0.16–0.75), and a regular bathing habit in river (AOR = 2.14, 95% CI 0.3–0.9) were independent predictors of helminth infections.

**Conclusion:**

Despite the fact that Ethiopia planned to eliminate helminth infection-related morbidity by 2020, this study showed that helminth infection is prevalent in the study area. Efforts should be made to improve hygienic practices of the schoolchildren in addition to school-based deworming. Moreover, the deworming program should also focus on reaching those SAC who do not attend school through communal social places to achieve the targeted goal in the study area in particular and nationwide in general.

## Background

Helminths are parasitic worms, widely distributed throughout the world causing large threats to the public health, economy, and physical and cognitive development particularly among SAC in developing countries [1, 2] and result in significant morbidity and mortality in endemic countries. The World Health Organization (WHO) estimates approximately 1.5 billion people are infected with soil-transmitted helminths (STHs) and that 207 million people currently have schistosomiasis, 90% of whom are living in Sub-Saharan Africa (SSA) [3, 4].

It is estimated that there are about 135, 000 deaths per year directly due to helminth infection around the globe [5]. Helminths are the main public health problem in many parts of the world. The principal public health significance of helminth infections lies in their chronic effects [6, 7]. The negative effect of helminth infection is common with heavy infection intensity in SAC [8]. Schistosomiasis and STHs are among the most widely distributed neglected tropical diseases affecting people living in tropical and developing countries [9]. Most infections with helminth occur in poor communities [10], where the biophysical, cultural, and environmental factors favor helminth transmission [11].

Low standard of living, poor personal hygiene, unsanitary waste management, and unsafe and inadequate water supply are some the factors that favor rapid transmission of helminths in developing countries [12]. Prevalence of STHs and *Schistosoma mansoni* (*S.mansoni*) across Africa estimated that Ethiopia to have the 13th highest prevalence of each diseases group among over 40 countries [13]. In Ethiopia, an estimated 37.3 million people are living with schistosomiasis, and 79 million live in schistosomiasis and STHs in endemic areas. Helminth infections constitute the greatest cause of illness and diseases in Ethiopia [14]. Several studies showed helminth infections due to *S.mansoni* and STHs are widely distributed in Ethiopia affecting mainly SAC [15–18]. Intestinal parasite infections including helminth infections are a significant health problem in Guragae zone. The overall prevalence of intestinal parasite infection was ranging between 38.5 and 40% before deworming is launched in the study area [19].

Currently, the primary control strategy for helminths mainly depends on mass drug administration (MDA) provided for the population at risk including SAC. WHO recommends MDA annually when the infection prevalence is in between 20 and 50% or bi-annually when the prevalence exceed 50% [9]. The national MDA program, launched since 2015 in Ethiopia, aims to eliminate STHs and schistosomiasis-related morbidity by 2020 [14]. Moreover, the MDA program goal in Ethiopia includes treating at least 75% of SAC in endemic districts [20]. Even though MDA does not prevent re-infection, repeated MDA has been known to have a considerable impact on maintaining the infection intensity of helminths to a minimal level and reducing the associated morbidities among infected individuals [21]. Therefore, this study aimed to determine the current status and infection intensity of helminth infections among SAC in the endemic area of Guragae zone, Southern Ethiopia, after 3 years of repeated deworming.

## Methods

### Study design and settings

An institution-based cross-sectional study was carried out between April to June 2017 in Gurage zone, Southern Nations, Nationalities, and People’s Regional State of Ethiopia. The zone is located at altitude ranging from 1600 to 2800 m above sea level. The mean maximum and minimum annual temperature of the zone is 25 °C and 7 °C, respectively.

### Study population and sampling technique

**Fig. 1:**
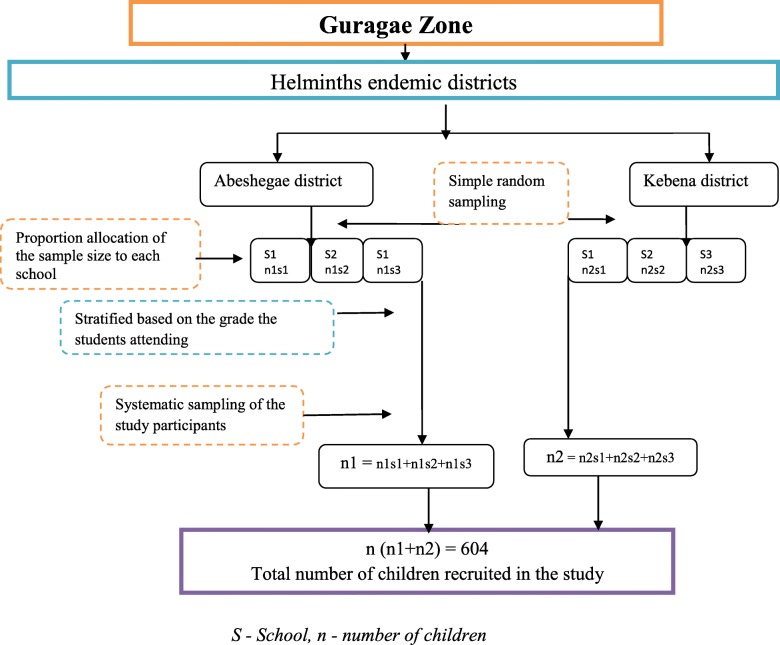
Flow chart of study participants sampling technique hierarchy

A single population proportion formula was used to calculate the sample size required for the study, with an assumption of the estimated prevalence of 23.3% [22], 95% confidence level, a desired precision of 5%, assuming additional 10% non-response rate, and a design effect of 2. Accordingly, the overall sample size calculated was 604 SAC. The proportionate number of study participant from each selected public elementary school was determined and each study subject was recruited using a systematic sampling technique as illustrated in Fig. 1.

### Inclusion and exclusion criteria

All children aged between 5 and 14 years children who were available in their school during data collection time were included. If selected children were not in class at the time of the survey visit, the next student on the sampling frame was considered and those who did not provide stool samples were excluded.

### Data collection procedure and data quality control

Data were collected using a semi-structured questionnaire. The questionnaire was initially prepared in English and then translated into Amharic and translated back into English to check its consistency by the principal investigator. A single fresh stool sample was collected from each study participants using clean, dry, and wide-mouthed labeled stool cups. Parasitological examination of stool samples was done in Wolkite University parasitology laboratory using the Kato-Katz egg counting technique. Briefly, the technique depends on microscopic counting of helminths and schistosome eggs in prepared slides with special staining of known weight of stool for helminth identification and enumeration as described elsewhere [23]. Infection intensities of the helminths were recorded and graded as light, moderate, or heavy based on the number of eggs per gram (EPG) of stool according to WHO threshold [24].

### Quality assurance

Data collectors were trained in data collection and laboratory procedure. Data collected using the questionnaire was checked for completeness at the end of each day. Duplicate Kato-Katz slides were prepared from each sample, and the slides were read by two different microscopists. Standard operating procedures were followed in every step of the laboratory analysis.

### Data processing and analysis

Data were entered, cleaned, and analyzed using SPSS for windows version 21 (Chicago, USA). The prevalence of helminths was calculated as the ratio of the number of children found positive for any helminth species to the total number of children who provided complete data. A co-linearity check was performed. Bivariate analysis was used to identify the associations of helminth infections with independent variables. All variables with *P* value less than 0.25 in the bivariate analysis were analyzed using multivariable logistic regression model. *P* values less than 0.05 were considered statistically significant.

### Study variables

The study variable includes sociodemographic characteristics (age, sex, residence, family unit size), family status (occupation of mother, educational status of the mother, occupation of father, educational status of the father), hygienic factors (shoe wearing, hand washing habit, habit of nail biting, habit of playing in the soil), source of water, and helminth infections.

## Results

### Demographic characteristics of the study participants

A total of 597 (98%) SAC was involved in this study. Age of the children ranged from 5 to 14 years with a median age of 10 years. The age group distribution showed that more than half (53.2%) were in the age group of 5–9 years. More than half (58.7%) of the children were males, and 59.1% of the households had a family size unit of between five and ten. About half (49.1%) of the children’s fathers and 46.3% mothers (guardians) had primary education while 63.1% of the children’s mothers and 65.1% children’s fathers were housewife and farmers in occupation, respectively(Table 1).

**Table 1:** Helminth infection in relation to sociodemographic characteristics among schoolchildren in the endemic area of Guragae zone, Southern Ethiopia, 2017

Variables	Total, *N* (%)	Helminth infection		COR (95% CI)	*P*	AOR (95% CI)	*P*
		Positive, *N* (%)	Negative, *N* (%)				
Age group in years							
5–9	318 (53.2)	77 (24.2)	241 (75.8)	1.2 (1.9–2.1)	0.03*	1.43 (0.4–0.9)	0.031*
10–14	279 (46.7)	52 (18.6)	227 (81.4)	1		1	
Gender							
Female	247 (41.3)	51 (20.6)	196 (79.4)	1			
Male	350 (58.7)	78 (22.2)	272 (77.8)	0.97	0.632	–	–
Fathers’ education							
No formal education	205 (34.3)	50 (24.3)	155 (75.7)	3.1	0.2	1.4 (0.1–19)	0.7
Primary	293 (49.1)	63 (21.5)	230 (78.5)	3.6	0.2	1.6 (0.1–22)	0.7
Secondary	80 (13.4)	10 (12.5)	69 (87.5)	6.2	0.08	2.6 (0.1–36)	0.4
Diploma and above	19 (3.2)	6 (31.5)	14 (68.5)	1		1	
Mothers’ education							
No formal education	276 (46.2)	60 (21.7)	216 (78.3)	1.3	0.2	1.6 (0.1–14)	0.6
Primary	277 (46.3)	61 (28.2)	216 (71.8)	1.3	0.3	1.4 (0.1–13)	0.7
Secondary	33 (5.5)	5 (15.1)	28 (84.9)	2.1	0.1	1.5 (0.1–15)	0.7
Diploma and above	11 (2.0)	3 (27.2)	8 (72.8)	1		1	
Mothers’ occupation							
Farmer	70 (11.7)	20 (28.5)	50 (77.5)	1			
Merchant	127 (21.3)	27 (21.2)	100 (78.8)	1.48	0.3		
Housewife	377 (63.10	79 (20.9)	298 (79.1)	1.5	0.32		
Others^b^	23 (3.9)	3 (13.1)	20 (86.9)	1.86	0.3		
Fathers’ occupation							
Farmer	389 (65.1)	95 (24.4)	294 (75.6)	1			
Merchant	95 (15.9)	16 (16.8)	79 (83.2)	1.6	0.1	1.4 (0.7–2)	0.2
Employed	63 (10.5)	11 (17.4)	52 (82.6)	1.5	0.1	1.3 (0.5–3)	0.5
Daily labor	37 (6.1)	4 (10.8)	33 (99.2)	2.6	0.07	2.4 (0.2–5)	0.1
Others^b^	13 (2.4)	3 (23.1)	10 (76.9)	1.6	0.5	1.06 (0.2–5)	0.9
Residence							
Urban	310 (51.9)	63 (20.3)	247 (79.7)	1			
Rural	287 (48.1)	66 (22.9)	221 (77.1)	1.17	0.4	–	–
Family size							
Less than 5	231 (38.6)	48 (20.7)	183 (79.3)	1			
Between 5 and 10	353 (59.1)	80 (22.6)	273 (77.4)	1.14	0.5	0.882 (0.5–1.3)	0.5
Greater than 10	13 (2.3)	1 (7.6)	12 (92.4)	3.14	0.2	3 (0.4–26)	0.2

*COR* crude odds ratio, *AOR* adjusted odds ratio, *CI* confidence interval, *P P* value, *N* number

*Statistically significant at *P* < 0.05

^b^Student, daily laborer

### Prevalence and infection intensity of helminth infections

Overall, at least one species of helminths was detected in 129 (21.6%) of the SAC, of which 12.9% (77/597) had *S.mansoni* (Table 3). Prevalence of helminth infection was associated with the age group of 5–9 years (*P* = 0.03). There was no significant difference of helminth infection with regard to gender (*P* = 0.97), mothers’ education (*P* = 0.7), family size (*P* = 0.5), and fathers’ and mothers’ occupation (*P* = 0.5) (Table 1). The most frequently encountered helminth was *S. mansoni* (12.9%) followed by hookworms (4.3%). The overall infection intensity of helminths expressed as geometric mean among the study participants for *Ascaris lumbricoides* (*A. lumbricoides)*, *Trichuris trichiura* (*T. trichiura)*, hookworms, and *S. mansoni* was 301, 31, 103, and 158 EPG, respectively. Most of the children had light infections, while 35.2% and 25.9% of children with *S.mansoni* infection had moderate and heavy infection intensity, respectively (Table 3).

### Factors associated with helminth infections

Children within the age group of 5–9 years (COR 1.2, 95% CI 1.3–2.1) and children whose family use river water for washing raw food and vegetables (COR 2.1, 95% CI 1.3–3.3) had significantly higher helminth infection (Table 2). Similarly, significantly higher helminth infection was observed in children with a habit of bathing in the river (COR 1.9, 95% CI 1.1–2.2) compared to those with a habit of bathing in the home using tap water (Table 2).

**Table 2:** Factors associated with helminth infection among schoolchildren in the endemic area of Guragae zone, Southern Ethiopia, 2017

Variables	Total, *N* (%)	Helminth infection		COR (95% CI)	*P*	AOR (95% CI)	*P*
		Positive, *N* (%)	Negative, *N* (%)				
Water for drinking							
From tap	539 (90.2)	114 (21.2)	425 (78.8)	1			
Protected well	45 (7.5)	13 (28.8)	32 (71.2)				
From river	11 (2.3)	2 (18.1)	11 (81.9)	4	0.99		
Water for washing raw food and vegetables							
From tap	380 (63.6)	67 (17.6)	313 (82.4)	1		1	
Unprotected well	62 (10.3)	14 (22.5)	48 (77.5)	2.1 (1.3–3.3)	0.001*	0.35 (0.1–0.8)	0.9
Protected well	9 (1.5)	2 (22.2)	7 (77.8)	1.5	0.196	1.03 (0.1–3.5)	0.5
From the river	146 (24.6)	46 (31.5)	100 (68.5)	1.6	0.562	2.4 (0.16–0.75)	0.008*
Fingernail status							
Trimmed	285 (47.7)	57 (20.0)	228 (80.0)	1		1	
Untrimmed	312 (52.3)	72 (23.1)	240 (76.7)	4	0.15	4.5 (0.3–1.2)	0.22
Shoe wearing habit							
Always	588 (98.4)	128 (21.4)	460 (78.6)	0.61	0.5		
Sometimes	9 (1.6)	1 (11.1)	8 (88.9)	1			
Hand washing habit after defecation							
Always	347 (58.1)	78 (22.4)	256 (77.6)	1			
Sometimes	250 (40.9)	51 (20.4)	202 (79.6)	1.01	0.9		
Hand washing habit before eating meal							
Always	218 (36.5)	51 (23.4)	167 (76.6)	0.001	0.9		
Sometimes	379 (63.5)	78 (20.5)	301 (79.5)	1			
Latrine availability							
Yes	566 (94.8)	121 (21.4)	445 (78.6)	1			
No	31 (5.2)	8 (25.8)	23 (74.2)	1.29	0.56		
Latrine type							
Flush latrine	94 (15.7)	23 (24.5)	71 (75.5)	1			
Pit Latrine	503 (84.3)	106 (17.8)	397 (82.2)	1.23	0.4		
Latrine utilization							
Latrine	490 (82.1)	103 (21.1)	387 (78.9)	1			
Open filled	107 (17.9)	26 (24.2)	81 (75.8)	1.22	0.4		
Bathing place							
Home	341 (57.1)	83 (24.3)	253 (75.7)	1		1	
River	179 (29.9)	28 (15.6)	151 (84.4)	1.9 (1.1–2.1)	0.009*	2.14 (0.3–0.9)	0.04*
Others	77 (13.0)	12 (15.5)	62 (84.5)	1.79	0.84		
Habit on soil playing							
Yes	315 (52.7)	122 (20.4)	249 (79.6)	1			
No	282 (47.3)	43 (15.2)	219 (84.8)	0.56	0.77		
Nail biting habit							
No	315 (52.7)	71 (22.5)	244 (77.5)	1			
Some times	282 (47.3)	58 (20.5)	224 (79.5)	2.1	0.21	1.3 (0.3–1.66)	0.1

*COR* crude odds ratio, *AOR* adjusted odds ratio, *CI* confidence interval, *P P* value, *N* number

*Statistically significant at *P* < 0.05

The multivariable logistic regression model estimated that children within the age group of 5–9 years were 1.43 times (AOR 1.43, 95% CI 0.4–0.9) more likely to get infected with helminths than those within the age group of 10–14 years(Table 1). Besides, the odds of helminth infection was 2.4 times (AOR 2.4, 95% CI 16–0.75) more for children whose family use river water for washing raw food and vegetables as compared to children’s family who uses tap water for washing raw food and vegetables. Children who had a regular bathing habit in the river were 2.1 times (AOR 2.1, 95%CI 0.3–0.9) more likely to get infected with helminths than children who had a regular bathing habit of in the home using tap water (Table 2).

## Discussion

Since Ethiopia launched the national MDA program in November 2015 [20], the control strategy in the country aims to eliminate STHs and schistosomiasis-related morbidity and to reduce the prevalence of heavy infection to less than 1% by 2020 [14]. The present study attempted to determine the status, infection intensity, and factors associated with helminths among SAC in Guragae zone. A survey provides pertinent information regarding the current status of helminths in the study area and enables evidence-based decision to be made in due course of the targeted elimination (Table 3).

**Table 3:** Infection intensity of schoolchildren infected with helminths in the endemic area of Guragae zone, Southern Ethiopia, 2017

Helminths	Number (%)	Geometric mean (EPG)	Light, no. (%)	Moderate, no. (%)	Heavy, no. (%)
Hookworms	26/597 (4.3)	103	26 (100)		
*Ascaris lumbricoides*	18/597 (3)	301	18 (100)		
*Trichuris trichiura*	3/597 (0.5)	31	3 (100)		
*Schistosoma mansoni*	77/597 (12.9)	158	30 (38.9)	27 (35.2)	20 (25.9)
*Others helminths	17/597 (2.8)	–			
Helminth co-infection	12/597 (2.1)	–			
Total	141/597 (23.6)	112			

*Enterobius vermicularis, Hymenolepis species

The fact that deworming does not prevent re-infection, repeated deworming has been known to have a considerable impact on maintaining the infection intensity of helminths to a minimal level, reducing the associated morbidities among infected individuals [25]. The overall intestinal parasite prevalence before the country-wide deworming program in the study area was ranging between 38.5 and 40% [19]. After 3 years of consecutive annual deworming, helminth infections have been prevalent in the study area. According to WHO STH and schistosomiasis endemic area classifications [9], the study area would be classified into the moderate transmission group and have been calling for annual deworming.

In Ethiopia, since the launch of the national MDA program, over 19 million people have been treated for schistosomiasis and the treatment coverage for schistosomiasis was 77% with 100% geographical coverage [14]. But, in the present study, *S.mansoni* was the most prevalent species of helminths and the majority of *S.mansoni*-infected children were classified under moderate to heavy infection intensity category. These may due to the impact of deworming depends on a variety of factors, including therapeutic efficacy of drugs used [26], the frequency at which deworming is given [27], individual factors, and the underlying intensity of parasite transmission [28]. In addition, the helminth transmission rate is influenced by environmental conditions that influence the survival and development of helminths free-living stages and socioeconomic factors which influence rates of exposure to infective stages [2].

Helminth infection is peaked in children aged between 4 and 8 years due to increased exposure with age and maintained constant possibly due to host resistance development and less exposure. However, there is no permanent development of protective immunity to re-infection [29]. In support of this, the prevalence of helminths in the present study showed significantly higher in age ranging from 5 to 9 years. A similar trend result was also observed in other studies conducted elsewhere [30, 31].

Washing raw food or vegetables using river water and bathing place were other key factors significantly associated with helminth infections in the present study. The finding is comparable with previous reports from the study area [19]. These results might indicate that using protected water and bathing place could contribute to a reduction in the prevalence of helminth infection. This highlighted that deworming should be backed up with health education to prevent re-infection for integrated control of helminths. Hence, increased coordination between deworming and health education program is essential to achieve the targeted elimination goals within the targeted time in the study area.

The present study had its own limitations; since only a single stool specimen was collected from each child, there might be an underestimation of the prevalence of helminths because of the temporal variation in egg excretion over hours and days. In addition, the study design being cross-sectional could underline the causal association between the helminths and socio-economic and demographic variables. Nevertheless, proportionate systematic sampling of the SAC from school would make it possible to generalize the finding to the whole SAC in the study area.

## Conclusions

Despite the fact that Ethiopia planned to eliminate helminth infection-related morbidity by 2020, the present study showed that helminth infection was prevalent among SAC in the study area. Moreover, poor personal hygiene such as regular bathing habit in the river and using river water for washing raw food and vegetables were the key factors significantly associated with helminth infections. Hence, efforts should be made to improve hygienic practices in addition to the school-based deworming program. The authors also believe that the deworming program should also focus on reaching those SAC who are not attending school through communal social places to achieve the targeted goal in the study area in particular and in the country in general.

## Abbreviations

EPG: Eggs per gram
MDA: Mass drug administration
SAC: School-aged children
SSA: Sub-Saharan Africa
STHs: Soil-transmitted helminths
WHO: World Health Organization
